# Köln-Timişoara Molecular Activity Combined Models toward Interspecies Toxicity Assessment

**DOI:** 10.3390/ijms10104474

**Published:** 2009-11-20

**Authors:** Sergiu A. Chicu, Mihai V. Putz

**Affiliations:** 1 Siegstr. 4, Köln, D-50859, Germany; E-Mail: Lchi@gmx.de (S.A.C.); 2 Laboratory of Computational and Structural Physical Chemistry, Chemistry Department, West University of Timişoara, Pestalozzi Street 16, Timişoara, RO-300115, Romania; Website: http://www.mvputz.iqstorm.ro

**Keywords:** Hydractinia echinata, *E*lement*S*pecific*I*nfluence*P*arameter-ESIP, Spectral-SAR, algebraic correlation, least action principle, Hansch parameters

## Abstract

Aiming to provide a unified picture of *computed* activity – quantitative structure activity relationships, the so called Köln (*ESIP-E*lement*S*pecific*I*nfluence*P*arameter) model for activity and Timisoara (Spectral-SAR) formulation of QSAR were pooled in order to assess the toxicity modeling and inter-toxicity correlation maps for aquatic organisms against paradigmatic organic compounds. The Köln ESIP model for estimation of a compound toxicity is based on the experimental measurement expressing the direct action of chemicals on the organism *Hydractinia echinata* so that the structural influence parameters are reflected by the metamorphosis degree itself. As such, the calculation of the structural parameters is absolutely necessary for correct evaluation and interpretation of the evolution of M(easured) and the C(computed) values. On the other hand, the Timişoara Spectral-SAR analysis offers correlation models and paths for *H.e.* species as well as for four other different organisms with which the toxicity may be inter-changed by means of the same mechanism of action induced by certain common chemicals.

## 1. Introduction

Directly and without delay inclusion of chemically artifacts in the biological cycle are due in the first line to solubility; from these, all less soluble, *i.e.*, those set down as sediments, suffer with the time various transformations with formation of new derivatives and with other possibilities of implication in the same natural biological cycle [1]. However, in the all of the cases the principal area of the accumulation is mainly the shallow marine water where the effects can be detected immediately to intimation or pursued in the time with different investigation methods.

*Hydractinia echinata*, as an organism living in the European and North-American coastal waters, could be directly affected by the presence of chemical derivatives through interruption of the evolution cycle at the level of the larva to polyp metamorphosis [2].

The testing of many anticonvulsants through which it was established that the order of influence is identical to that obtained through treatment of the embryo *in vitro* [3] was first achieved by use of the *Hydractinia echinata* metamorphosis stage for monitoring toxicity problems. The research continued by establishing various relationships between structure and reactivity of oil and oil products, alkanes, cycloalkanes, aromatic compounds [4], as well as for a series of hydrocarbon derivatives, aliphatic alcohols, aliphatic amines, aminoalcohols [5], or phenols[6].

The *Hydractinia echinata* test system was already demonstrated to be applicable for very different series of derivatives or products, including pharmaceutical products for dentistry, natural extracts, detergents, dyes, etc. Their interactions on living cells from measured (M) values for simple organic molecules by means of the introduced *ESIP-*parameters (*E*lement*S*pecific*I*nfluence*P*arameter) models the molecular substructures for their computed (C) toxicity of containing substances represent the essence of the so called “Köln model” [5]. On the other side the recently developed so called *Spectral-SAR* as the “Timisoara QSAR model” allows for mechanistic description of the molecular specific actions throughout combined reactivity-activity paths of interactions [7–10].

In this context, the present endeavor combines ESIP and S-SAR models for advancing a sort of “absolute” analysis of ecotoxicity employing the computed activities of their spectral correlation, respectively, for an inter-species analysis for a common set of compounds. As such, having at hand a complex method providing both the organisms’ toxicity activity (ESIP), without the need to undertake extensive experiments for measuring them, as well as the mechanistically revealed path of molecular action (Spectral-SAR) may constitute an advancement in ecotoxicological assessments through computational design and reasoning. This way, the *in silico* methods will eventually reveal the mechanisms of toxicity for a given set of toxicants and environmental hazards, while lowering the experimental costs.

## 2. Background Models

### 2.1. Köln ESIP Model for Biological Activity

We determinate *ESIP*-parameters based on the measured values basis Mlog(1/MRC_50_) [Mol/L] in order to calculate toxicity values Clog(1/MRC_50_) [Mol/L] for untested derivatives [5]. The molecular structures have always saturated hydrocarbon or aromatic substructures, so the first *ESIP*-parameter corresponds to saturated-carbon *ESIP*c-sat, followed by the aromatic-carbon *ESIP*c-ar, and *ESIP*-organic function (alcohol, amino, etc.). In the case of saturated hydrocarbons the *ESIP*c-sat have an average value of 0.50 log units, calculated on the basis of measured values M and saturated carbon numbers C.

In this way, the toxicity of not tested compounds can be calculated with the following assumptions:

- the toxicity of a compound can be subdivided into that of components (*ESIP*’s) in such a way that the sum of these components results in the total toxicity value;
- these components (*ESIP*’s) are identical in different substances;
- the *ESIP*’s components have a dynamical value (they depend on the determined number or are derived from newly available data) for one organism and a test-system, while varying for different test-systems. However, if a deviation between the measured M and the calculated C values is observed, there is an indication of an overlooked interaction between different parts of the molecule, or may indicate an activity of a substance specific for a certain biochemical pathway.

Note that somewhat similar studies were examined at the inter-species toxicity level by the aid of data bases centered on a given species [11], although this limits the possibility to dynamically extend the molecular group toxicity from one organism to other [12,13], as the *ESIP* method is able to do.

### 2.2. Timişoara Spectral-SAR Model

Since QSAR models aim at correlations between concerned (congener) molecular structures and measured (or otherwise evaluated) activities, it appears naturally that the *structure* part of the problem be accommodated within the quantum theory and of its formalisms. In fact, there are few quantum characters that we are using within the present approach:

- ○ Any molecular structural state (dynamical, since undergoes interactions with organisms) may be represented by a | *ket*〉 *state vector*, in the abstract Hilbert space, following the 〈*bra | ket*〉 Dirac formalism [14]; such states are to be represented by any reliable molecular index, or, in particular in our study by hydrophobicity |*LogP*〉, polarizability |*POL*〉, and total optimized energy |*E_tot_*〉, just to be restrained only the so called Hansch parameters, usually employed for accounting the diffusion, electrostatic and steric effects for molecules acting on organisms’ cells, respectively.
- ○ The (quantum) *superposition principle* assuring that the various linear combinations of molecular states map onto the resulting state, here interpreted as the bio-, eco- or toxico-logical activity, *e.g.*, |*Y*〉 = |*Y*_0_〉 +*C_LogP_* |*LogP*〉 +*C_POL_* |*POL*〉 +..., with |*Y*_0_〉 meaning the free or unperturbed activity (when all other influences are absent).
- ○ The *orthogonalization feature* of quantum states, a crucial condition providing that the superimposed molecular states generates *new* molecular state (here quantified as the organism activity); analytically, the orthogonalization condition is represented by the 〈*bra* | *ket*〉 scalar product of two envisaged states (molecular indices); if it is evaluated to zero value, *i.e.*, 〈*bra* | *ket*〉 = 0, then the convoluted states are said to be orthogonal (zero-overlapping) and the associate molecular descriptors are considered as independent, therefore suitable to be assumed as eigen-states (of a *spectral* decomposition) in the resulted activity state, while quantified by the degree their molecular indices enter the activity correlation. Further details on scalar product and related properties are given in Appendix A1, whereas in what follows the Spectral-based SAR correlation method (thereby called as Spectral-SAR) is resumed.

Note that since molecular states are usually represented by *ket* vectors which are a generalization of custom (classical) vectors, all formalisms are consistently developed accordingly. In this regard, the *bra-ket* formalism is more than a simple notation – it is indeed a reliable formalism since, for instance, it differentiates between the dual and direct spaces the *bra*- and *ket*- vectors are attributed to, respectively, with insightful consequences for the space-time evolution of a system – a matter not conveyed by classical simple vectorial notation. However, it is not a complication of reality but a close representation of it: the molecular descriptors belong to a given molecular state that *has* to be included as a component of the quantum (*ket*) vectors carrying the specific structural information – a feature not fulfilled by simple classical vectors. Therefore, the adopted vectorial formalism goes beyond the simple notation – each time when we write a *ket* vector represented by a structural index we see in fact a generalized electronic (for a hyper-molecular) state, defined as the global state collecting one descriptor’ values for all concerned congener molecules.

Now, a set of *N* molecules studied against observed/recorded/measured biological activity is represented by means of their *M* – structural indicators (the states); all the *N* × *M* input information may be expressed by the vectors-columns of the Table 1 and correlated upon the generic scheme of Equations (1a)–(1d):

(1a) $$|Y_{\text{OBS}\;(\text{ERVED})}\rangle =|Y_{\text{PRED}\;(\text{ICTED})}\rangle +|\text{prediction}\;\text{error}\rangle$$

(1b) $$=b_{0}|X_{0}\rangle +b_{1}|X_{1}\rangle +\ldots +b_{k}|X_{k}\rangle +\ldots +b_{M}|X_{M}\rangle +|\text{prediction}\;\text{error}\rangle$$

where the vector |*X*_0_〉 = 1 1 ... 1*_N_*〉was added to account for the free activity term.

In order for equation (1b) to represent a reliable model of the given activities, the hyper-molecular states (indices) assumed should constitute an orthogonal set, having this constraint a consistent quantum mechanical basis, as above described. However, unlike other important studies addressing this problem [15–17], the present Spectral-SAR [7] assumes the prediction error vector as being orthogonal to all others:

(1c) $$\langle Y_{\text{PRED}}|\text{prediction}\;\text{error}\rangle =0$$

since it is not known *a priori* any correlation is made. Moreover, Equations (1a), (1b), and (1c) imply that the prediction error vector has to be orthogonal on all known descriptors (states) of predicted activity:

(1d) $$\langle X_{i=\bar{0,M}}|\text{prediction}\;\text{error}\rangle =0$$

assuring therefore the reliability of the present *ket* states approach. In other terms, conditions (1c) and (1d) agree with Equation (1a) in the sense that the prediction vector and the prediction activity *Y_PRED_* (with all its sub-intended states $|X_{i=\bar{0,M}}\rangle$) belong to disjoint (thus orthogonal) Hilbert (sub)spaces; or, even more, one can say that the Hilbert space of the observed activity |*Y_OBS_*〉 may be decomposed into a predicted and error independent Hilbert sub-spaces of states.

Therefore, within Timişoara Spectral-SAR procedure the very first step consists in orthogonalization of *prediction error* on the predicted activity and on its predictor states, while the remaining algorithm does not seek to optimize the minimization of errors, but for producing the ideal correlation between |*Y_PRED_*〉 and the given descriptors $|X_{i=\bar{0,M}}\rangle$.

Next, the Gram-Schmidt orthogonalization scheme is applied through construction of the appropriate set of descriptors by means of the consecrated iteration [16,18,19]:

(2a) $$|\Omega_{0}\rangle =|X_{0}\rangle$$

(2b) $$|\Omega_{k}\rangle =|X_{k}\rangle -\sum_{i=0}^{k-1}r_{i}^{k}|\Omega_{i}\rangle$$

(2c) $$r_{i}^{k}=\frac{\langle X_{k}|\Omega_{i}\rangle }{\langle \Omega_{i}|\Omega_{i}\rangle },k=\bar{1,M}$$

providing the orthogonal correlation:

(3a) $$|Y_{\text{PRED}}\rangle =\omega_{0}|\Omega_{0}\rangle +\omega_{1}|\Omega_{1}\rangle +\ldots +\omega_{k}|\Omega_{k}\rangle +\ldots +\omega_{M}|\Omega_{M}\rangle$$

(3b) $$\omega_{k}=\frac{\langle \Omega_{k}|Y_{\text{PRED}}\rangle }{\langle \Omega_{k}|\Omega_{k}\rangle },\;k=\bar{0,M}$$

Remarkably, while available studies dedicated to the orthogonality problem usually stop at this stage, the Spectral-SAR uses it to provide the solution for the original sought correlation of Equation (1b) – having the prediction error vector orthogonal to the predicted activity and all its predictor states of Table 1. This can be wisely achieved through grouping Equations (2) and (3) so that the system of all descriptors of Table 1 is now written in terms of orthogonal descriptors:

(4) $$\{\begin{array}{l} |Y_{\text{PRED}}\rangle =\omega_{0}|\Omega_{0}\rangle +\;\;\;\;\omega_{1}|\Omega_{1}\rangle +\ldots +\;\;\omega_{k}|\Omega_{k}\rangle +\ldots +\omega_{M}|\Omega_{M}\rangle \\ |X_{0}\rangle \;\;\;\;\;\;\;=1.|\Omega_{0}\rangle +\;\;\;\;0.|\Omega_{1}\rangle +\ldots +\;\;0.|\Omega_{k}\rangle +\ldots +\;\;0.|\Omega_{M}\rangle \\ |X_{1}\rangle \;\;\;\;\;\;\;=r_{0}^{1}|\Omega_{0}\rangle +\;\;\;\;1.|\Omega_{1}\rangle +\ldots +0.|\Omega_{k}\rangle +\ldots +0.|\Omega_{M}\rangle \\ ........................................................................................\\ |X_{k}\rangle \;\;\;\;=r_{0}^{k}|\Omega_{0}\rangle +\;\;\;r_{1}^{k}|\Omega_{1}\rangle +\ldots +1.|\Omega_{k}\rangle +\ldots +0.|\Omega_{M}\rangle \\ ........................................................................................\\ |X_{M}\rangle \;\;\;=r_{0}^{M}|\Omega_{0}\rangle +\;\;\;r_{1}^{M}|\Omega_{1}\rangle +\ldots +r_{k}^{M}|\Omega_{k}\rangle +\ldots +1.|\Omega_{M}\rangle \end{array}$$

According with a well known algebraic theorem, the system (4) has no trivial solution if and only if the associated extended determinant vanishes; this way the Spectral-SAR determinant features the form [7]:

(5) $$\left|\begin{array}{lllllll} |Y_{\text{PRED}}\rangle & \omega_{0} & \omega_{1} & \ldots & \omega_{k} & \ldots & \omega_{M}\\ |X_{0}\rangle & 1 & 0 & \ldots & 0 & \ldots & 0\\ |X_{1}\rangle & r_{0}^{1} & 1 & \ldots & 0 & \ldots & 0\\ \ldots & \ldots & \ldots & \ldots & \ldots & \ldots & \ldots \\ |X_{k}\rangle & r_{0}^{k} & r_{1}^{k} & \ldots & 1 & \ldots & 0\\ \ldots & \ldots & \ldots & \ldots & \ldots & \ldots & \ldots \\ |X_{M}\rangle & r_{0}^{M} & r_{1}^{M} & \ldots & r_{k}^{M} & \ldots & 1 \end{array}\right|=0$$

Now, when the determinant of Equation (5) is expanded on its first column, and the result is rearranged so that to have |*Y_PRED_*〉 on left side and the rest of states/indicators on the right side the sought QSAR solution for the initial observed-predicted correlation problem of Equation (1a) is obtained under the Spectral-SAR vectorial expansion (from where the “spectral” name is justified) without the need to minimize the predicted error vector anymore, being this stage absorbed in its orthogonal behavior with respect to the predicted activity.

In fact, the Spectral-SAR procedure uses the double conversion idea: one forward, from the given problem of Equations (1a)–(1d) to the orthogonal one of Equation (3) in which the error vector has no manifestation; and a backwards one, from the orthogonal to the real descriptors by employing the system (4) determinant (5) expansion as the QSAR solution.

It is worth stressing that the present QSAR/Spectral-SAR equations are totally delivered from the (analytical) determinant (5) and not computationally restricted to the inverse matrix product as prescribed by the fashioned statistical Pearson approach [20]. Moreover, the Spectral-SAR algorithm is invariant also upon the order of descriptors chosen in orthogonalization procedure, providing equivalent determinants no matter how its lines are re-derived, an improvement that was not previously achieved by other available orthogonalization techniques [15,17].

However, besides the effectiveness of the S-SAR methodology in reproducing the old-fashioned multi-linear QSAR analysis [7,21], one of its advantages concerns on the possibility of introducing the so called (*vectorial*) *norms* (see Appendix A1) associated with either *experimental* (measured or observed) or *predicted* (computed) activities:

(6) $$\left|\left||Y_{\text{OBS}/\text{PPED}}\rangle \right|\right|=\sqrt{\langle Y_{\text{OBS}/\text{PRED}}|Y_{\text{OBS}/\text{PRED}}\rangle }=\sqrt{\sum_{i=1}^{N}y_{i-\text{OBS}/\text{PRED}}^{2}}$$

They provide a unique assignment of a number to a specific type of correlation, *i.e.*, by performing a sort of final quantification of the models. Nevertheless, the activity norm given in Equation (6) opens the possibility of replacing the classical statistical correlation factor [21]:

(7) $$R\equiv r_{\text{STATISTIC}}=\sqrt{1-\frac{\sum_{i=1}^{N}{\left(y_{i-\text{OBS}}-y_{i-\text{PRED}}\right)}^{2}}{\sum_{i=1}^{N}{\left(y_{i-\text{OBS}}-\frac{1}{N}\sum_{i=1}^{N}y_{i-\text{OBS}}\right)}^{2}}}$$

with a new index of correlation, introduced as the so called *algebraic S-SAR correlation factor* (or *R*-algebraic, shorthanded as *RA*) through the ratio of the predicted to observed norms [22,23]:

(8) $$RA\equiv r_{\text{ALGEBRAIC}}=\sqrt{\frac{\sum_{i=1}^{N}y_{i-\text{PRED}}^{2}}{\sum_{i=1}^{N}y_{i-\text{OBS}}^{2}}}=\frac{\left|\left||Y_{\text{PRED}}\rangle \right|\right|}{\left|\left||Y_{\text{OBS}}\rangle \right|\right|}$$

It has the meaning of realization probability with which a certain predicted model approaches the observed activity throughout all of the employed molecules (in the hyper-molecular states of activities), see Appendix A2.

With this interpretation the algebraic correlation conceptually departs from the statistical one in that the later accounts on the degree with which each computed individual molecular activity approaches the *mean* activity of the *N*-molecules, while the first evaluates the (hyper-molecule) degree of overlap of predicted to observed activities’ norms (viewed as the “amplitudes” of molecular-organism interaction’s intensity). In this respect there seems that the algebraic analysis is more suited to environmental studies in which the *global* rather than *local* effect of a series of toxicants is evaluated on specific species and organisms.

In fact, this new correlation factor definition compares the vectorial lengths of the predicted activity against the measured one, thus being an indicator of the extent with which certain computed property or activity approaches the “length” of the observed quantity.

However, it was already shown that the algebraic correlation factor of Equation (8) furnishes higher and more insightful values than its statistical counterpart in a systematical manner [21,24], thus advancing it as the ideal tool for correlation analysis on a shrink interval of data analysis where the statistical meaning is naturally lost.

Even more, in the terms of the “quantum spectral” formalism, one can say that algebraic investigation provides the “excited” states of an activity modeling, while the statistical approach deals with “ground state” or lower states of correlation. Consequently, for completeness, a proper quest of structure-activity models should include both of these stages of molecular SAR modeling.

Going further towards extracting the mechanistic information from the Spectral-SAR norms and correlation factors we can further advance the so called *least path principle*:

(9) $$\delta [A_{1},A_{2},\ldots ,A_{M}]=\delta \left(\sum_{i=1}^{M-1}\left[A_{i},\;A_{i+1}\right]\right)=0$$

applied upon successively connected models with different correlation dimensions: it starts from 1-dimension with a single structural indicator correlation, say *A*_1_, until the models with maximum factors of correlation, say *A_M_* – *i.e.*, containing *M* number of indicators, see Table 1) [7–10]. Since each of these models is now characterized by its predicted activity norm ‖|*Y_PRED_*〉‖ along the algebraic (*RA*) and/or statistical (*R*) correlation factors, the elementary paths of Equation (9) are constructed as the Euclidian measure between two consecutive models (endpoints) [7–10,22–24]:

(10) $$[A_{1},\;A_{2}]=\sqrt{{\left(\left|\left||Y_{\text{PRED}}^{A_{2}}\rangle \right|\right|-\left|\left||Y_{\text{PRED}}^{A_{1}}\rangle \right|\right|\right)}^{2}+{\left(r_{\begin{array}{l} \text{ALGEBRAIC}\\ \text{STATISTIC} \end{array}}^{A_{2}}-r_{\begin{array}{l} \text{ALGEBRAIC}\\ \text{STATISTIC} \end{array}}^{A_{1}}\right)}^{2}}$$

It is noteworthy that the formal equation (9) has to be read as searching for paths’ combination on the left side providing minimum value in the right side; it is practiced as the tool for deciding the hierarchy along all (ergodic) possible end-point linked paths with the important consequence of picturing the mechanistic and causal evolution of structural influences that trigger the observed effects.

This methodology was successfully applied in ecotoxicology [7,8,24] and for designing the behavior of the species interactions within a test battery [23], promising to furnish adequate framework also for the present (and future) interspecies analysis.

## 3. Spectral-SAR Results

Data of Table 2 are modeled as QSARs for each species in both Mlog and Clog modes, with the help of Spectral-SAR determinant (5), wile reporting the algebraic norms and correlation computed upon Equations (6) and (8), respectively, side-by-side with the statistical correlation coefficients of Equation (7). The results are listed in Tables 3 and 4 for employed Mlog and Clog-*ESIP* data of Table 2, respectively. However, in order to assure the reliability for the computed models the so called Topliss-Costello rule was considered, *i.e.*, building models with about five times ratio of activity points with respect to the number of correlating/structural variables [25].

Aiming to provide the mechanistic maps of actions for the targeted species, the minimization principle of Spectral paths given by Equations (9) and (10) is considered among all possible ways of connecting endpoints from each category of models (*i.e.*, with one, two or three dependency factors). The Tables 5 and 6 present all these endpoints’ paths for Mlog and Clog activities, computed upon Equations (6)–(8) and (10) through processing the data of Tables 3 and 4, respectively.

However, in order to identify the shortest paths in each category of endpoint connections, according with prescription given by Equation (9), the following rules are applied:

- the first choice is the overall minimum path, in a certain column of Tables 5 and 6 (either for statistical or algebraically correlation);
- if the overall minimum is reached by many equivalent paths (as is the case of Mlog-algebraic column for *H.e.* in Table 5, for instance) the minimum path will be considered that one connecting the starting endpoint with the closest endpoint in the sense of norms (as is for *H.e./* Mlog the norm of |2> state the closest to the norm of |2,3> state, as compared with |1,2> and |1,3>, see Spectral-SAR norm column of Table 3, for example);
- the overall minimum path will set the dominant hierarchical path in assessing the mechanistically mode of action towards the given/measured activity; it is called as *the alpha path* (*α*);
- once the alpha path has been set the next minimum path will be looked for in such a way that the new starting endpoint is different from that one already involved in the alpha path (that is, if in the established *alpha* path for *H.e./* Mlog the starting model correspond to the |2> state, the next path to be identified will originate either on models/states |1> or |3>);
- the remaining minimum paths are identified on the same rules as before and will be called like *beta* and *gamma paths*, *β* and *γ*, respectively;
- at the end of this procedure each mode of action is to be “touched” only one, excepting the final endpoint state {|1,2,3>} that can present *degeneracy*, *i.e.*, may be found with the same influence at the end of various paths, herein called as *degenerate paths* (*e.g.*, the states |1,2,3>, |2,1,3>, and |3,1,2> in the case of *Hydractinia echinata* and *Tetrahymena pyriformis* at their ending toxicity paths of Table 5); Yet, such behavior may leave with the important idea the *degenerate paths*, although different in the start and intermediate states, while ending with the same ordering influences, *e.g.*, the state |2,1,3> of Table 5 (with “1” for LogP, “2” for POL, and “3” for E_tot_, see Tables 3 and 4), provides weaker contribution to the recorder activity since two paths have to produce the same (final) effect in order it to be activated; this is nevertheless one remarkable mechanistic consequence of the present combined (algebraic or statistical) correlations with minimization (optimization) principle applied for the spectral path lengths through Equations (6)–(10);
- the *alpha*, *beta* and *gamma* paths can be easily identified for algebraic and statistical treatments in Tables 5 and 6 and there are accordingly marked; the degeneracy behavior is readily verified in Table 5 where the *alpha path is found as the only (non-degenerate) path* out of all possible ones. Of course, the same rationalization applies also for *alpha* path of Table 6, however displaying the trivial situation in which the absence of any degeneracy is recorded due to the restrained structural parameters considered for activity modeling since less available data for *Pimephales promelas* (*P.p.*) and *Vibrio fisheri* (*V.f.*) species in Table 2, according with the above specified Topliss-Costello rule.

Now, the *interspecies* analysis may be unfolded employing the paths of Tables 5 and 6; for achieving that, a preliminary search for minimum paths at the *inter*-species levels for each Mlog/Clog and algebraic/statistic computational frames should be done first.

**Figure 1.**
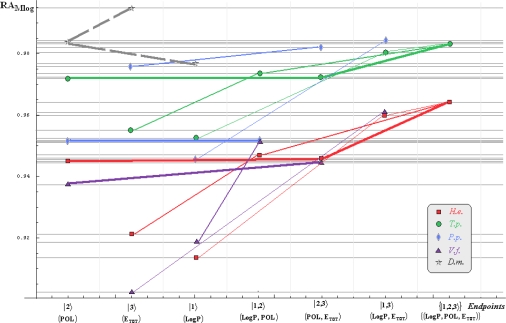
The *Hydractinia echinata* (*H.e.*), *Tetrahymena pyriformis* (*T.p.*), *Pimephales promelas* (*P.p.*), *Vibrio fisheri* (*V.f.*), and *Daphnia magna* (*D.m.*) interspecies Spectral-SAR map modeling the molecular mechanisms for Mlog-algebraic toxicity paths of Tables 5 and 6 connecting the algebraic correlations of Table 3 across the ordered models of Table 7; the difference between species is made by the assignments of distinct icons, while *alpha*, *beta* and *gamma* paths are differentiated by thickness decreasing of lines joining the same icons; the *D.m.* pseudo-path (interrupted line on map) is considered from the highest correlation model towards the lowest one in Table 3.

**Figure 2.**
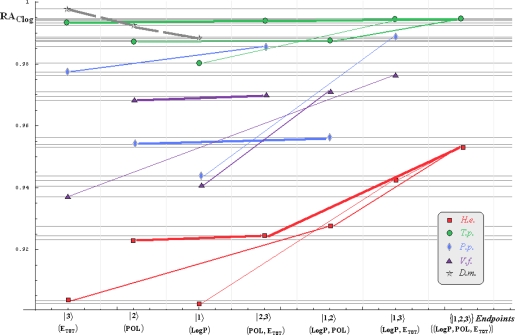
The same type of representation as of Figure 1, here at the Clog-algebraic level.

**Figure 3.**
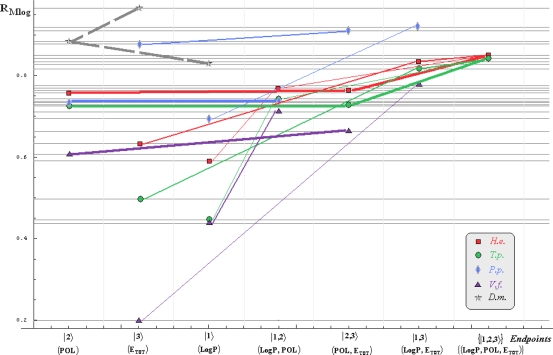
The same type of representation as of Figure 1, here at the Mlog-statistic level.

**Figure 4.**
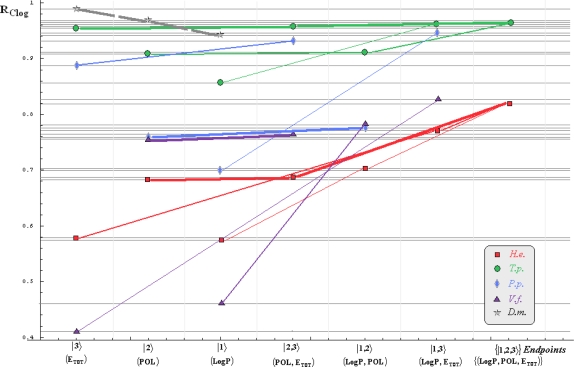
The same type of representation as of Figure 1, here at the Clog-statistic level.

Note that for *Daphnia magna* (*D.m.*) species, although specific paths would be superfluous with the uni-parameter models considered in Tables 3 and 4 due the Topliss-Costello rule (since the limited data of Table 2), its presence on the inter-species grids of Figures 1–4 may be as well considered by means of the *pseudo-path construction* based on reconsidering the above a) & b) minimum searching rules for models with single parameter dependency:

h) models with higher correlation/probability (either within statistic or algebraic approaches) will firstly enter molecular mechanism of toxicity through their considered structural parameter, *i.e.*, LogP, POL and E_tot_ for the |1>, |2> and |3> end-points, respectively.

Such a quest is performed in two steps: the computational scheme is primarily fixed, *e.g.*, the Mlog-algebraic one; then, among all Mlog-algebraic *alpha* paths for all species of Tables 5 and 6 the minimum is selected, *i.e.*, *α_P.p._* for the actual case.

Then, the same procedure is unfolded for the remaining *beta* and *gamma* paths within the fixed computational frame, *i.e.*, it will be repeated for each possible Mlog/Clog-algebraic/statistic combination. The results are summarized in Table 7 leading with the interspecies ordering of models to be considered for a mechanistic Spectral-SAR analysis. As such, all possible inter- and intra-species influences are presented in Figures 1–4 emphasizing on primary (*alpha*), secondary (*beta*) and tertiary (*gamma*) paths of Tables 5 and 6 projected on the Mlog/Clog models for algebraic/statistic correlations of Tables 3 and 4, respectively.

The inter-species diagrams reveal interesting features respecting both the correlation analysis and the inter-toxicity; as such, when is about either of algebraic or statistical treatment either Mlog- and Clog- interspecies ecotoxicity diagrams display the same endpoint ordering, as revealed by Table 7 and Figures 1 & 3 and 2 & 4, respectively. Beyond this, the algebraic approaches provide better systematic maps of inter-toxicity judged upon the *minimum distribution of crossing individual species’ paths* (*alpha*, *bet*a or *gamma*), being this another realization of the least path principle – here at inter-species paths’ level; for instance, the *H.e.* paths within algebraic framework RA_Clog_ of Figure 2 are clearly individuated as having no crossing toxicity with other species eventually submersed in the same ecological area, while the carried toxicity may be transmitted to *V.f.* species according with the statistical approach R_Clog_ of Figure 4.

On the other way, when comparing the measured (observed) results it is apparent that the species *H.e.* and *V.f.* are eco-toxically interconnected and somehow independent from the *T.p.* and *P.p.* environmental response in RA_Mlog_ picture of Figure 1. Yet, a different situation is noted for the statistical R_Mlog_ analysis of Figure 3, according which *H.e.* species is highly mixed from a toxicological point of view with the species *T.p.* and *P.p.*, but not with the *V.f.* one, either by means of first (*alpha*), second (*beta*) or third (*gamma*) toxicity paths.

Finally, the species *D.m.* is predicted to strongly interact (crosses at the *alpha* paths’ level) with the species *T.p.* on both algebraic RA_Clog_ and statistical R_Clog_ frameworks of Figures 2 and 4 due to POL and LogP parameters specific influence - identified on the grid region of their path crossings, respectively. Such a situation is no longer valid when Mlog values are modeled, since the algebraic RA_Mlog_ approach predicts moderate inter-toxicity influence (through *alpha-beta* crossing paths due E_tot_ or steric influence) (see Figure 1), in contrast with no recorded interaction within the statistical R_Mlog_ analysis (see Figure 3).

Therefore, the molecular mechanistic models of toxicity may be proposed in four variants: based on algebraic (RA) or statistic (R) correlation of either measured (Mlog) or by *ESIP* computed (Clog) toxicities.

The difference between the algebraic and statistical approaches relays on their inner definition: while, for a data sample, the statistical framework quantifies the dispersion respecting the data average (the data mean), the algebraic picture accounts for the dispersion of the extremes (the *N*-dimensional Euclidian lengths of the data rows); from this conceptual difference, although both assess the same confined realm between 0 and 1 in probability realization, the algebraic correlation records closer values near to the certainty for models classified as with high or even moderate statistical correlation values [7], being thus more suited for *least path principle* applications, as also proven by the current study.

In other words, if one is interested in the sample data behavior merely from its “length” (the norm) than from its “average dispersion” side, the algebraic way should be chosen as the main correlation framework, while keeping the statistical counterpart available for comparison purpose. This seems to be the case of ecotoxicological studies when the intensity (the “length or the amplitude”) of action for each sample’s endpoint may be important [8].

On the other hand the difference between the measured and computed values for ecotoxicological activities relays on the way the *ESIP* model is build from the available database, *i.e.*, by collecting the measured molecular-upon-species values and then appropriately redistributing them among various molecular fragments and groups of actual interest. Nevertheless, a practical discussion on how fine the actual *ESIP* data accommodates with the correlated structural data is addressed next.

## 4. Discussion of ESIP

Table 2 presents the 28 tested combinations of *H.e.* with other organisms. In the case of derivatives nos. 1–25, the estimations of the calculated (C) values have been possible through use of the *ESIP*’s parameters distinctive for molecular substructures and specifically for every test-system. The file (structure + *ESIP* algorithm) of the mentioned derivatives offers the possibility of the analysis of different structure-reactivity relations through mentioned organisms we follow.

In the *H.e*. case, a specifically marine environment organism, pure water has the least toxicity and has the least values for structural parameters. The appearance of the hydrocarbonated chain leads to increasing molecular toxicity simultaneously with the increased values of the structural parameters (logP and POL) in the case of alcohols (nos. 2–4, and 6) and in the case of the phenols (nos. 14, 15, and 21) too. Compound no. 21 has the highest toxicity, probably due to geometry of the hydrocarbon radical situated in opposite *para*- position for phenolic hydroxyl and this one proximity on aromatic nucleus [27]; It is worth observing that the steric impediments limit produces such increase in the case of 2,6-diisopropylphenol. Instead, 1,2,3-propanetriol possess three OH groups, leading with persistent hydrophilic character, while the molecule has a diminished toxicity according to the logP value.

In the aromatic series (molecules nos. 16, 18, and 20) the structural parameters have closer values but the toxicities are more elevated as a result of the possibilities of extended electronic conjugation. The existence of two identical hydroxyl groups (see molecule no. 18), a highly symmetrical and flat molecule, as well as the absence of sterical hindrances, are considered to be the premises of an extended p-π conjugation (the possibility of conjugation between the non-bonded p electrons of Oxygen and the π electrons of aromatic centre) according to a push-pull electronic mechanism: an OH group is electron donating and becomes positively-charged, and the second one, an electron accepting group becomes negatively-charged. This phenomenon, which is probably alternant and permanent even in the absence of a reaction partner, induces a strong hydrogen bond donor character. Unexpected seems the toxicity of 1,2,3-trihydroxibenzene (molecule no. 20), essentially identical with that of 1,2-dihydroxybenzene, although its logP value is diminished; this maybe happens due the push-pull mechanism of polyhidroxylic phenols [6].

In the case of 1,4-dihydroxybenzene, the conjugation is diminished through the inclusion of a *t*-butyl radical (no. 19) and increased steric impediment at the phenolic hydroxyl level. However, the toxicity significantly decreases by three orders by replacing one −OH group with a methyl (no. 14), methoxy (no. 17), chloro (no. 22) or amino (no. 24) moiety though the logP parameter changes significantly. The situation according to which the toxicity values of the mentioned derivatives span a narrow domain of about 0.5 logarithm units, relays on the existence of certain stereo-electronic balance similarity in the case of aromatic derivatives inferior substituted, as 4-toluidine (no. 11) and 1,2-dichlorobenzene (no. 12), in agreement with other (unpublished) series of derivatives.

The toxicity dependence on the logP parameter is informative also for derivatives with pyridine nitrogen (no. 8–10), quinonic ones (no. 26 and 27) and 2,4,6-trinitrophenol (no. 25), which although having the most diminished logP value, displays however identical efficacy with monosubstituted phenol derivatives.

An example of the influence of functional groups is illustrated by the 1,10-diaminodecane (no. 7) molecule with a smaller toxicity than expected according to its hydrocarbonated chain, though the POL value is great. According with the experimental results [5], the chain with 8–10 C probably represents the hydrocarbon interface where the lipophilicity is manifested. Yet, the diffusion of the molecules with high number of the C atoms through the cell membrane is however “hindered” and the percentage of the crossing molecules is diminished [28].

The presence of the 4-methoxyazobenzene (no. 28) in Table 2 illustrates that as profound structural changes resulted new reactions mechanisms appear, emphasizing the availability offered by the test-system with *H.e.* to analyze very different derivatives. This is the case of the azo-function which, by means of enzymatic reduction, leads to the stoichiometric appearance of amines, though the toxicity of the mixed combination (the most frequent case in the environment) represents a fruitful and significant investigation direction.

The *ESIP*-Köln model provides, although not in all cases, the possibility to appreciate to a great extent the efficiency with which the real or measured M value agrees with the theoretically calculated (C) counterparts. In other words, if the calculated value (C) stands above those measured (M), for instance, the difference can be assigned to the lipophilicity character represented here by logP, along some electronic POL or steric Etot influences.

However, the *ESIP* values have been determined to bend down on real/measured values through inclusion of the effects relating specific parameters of molecular structures. This is confirmed since the measured Mlog/MRC_50_ and calculated Clog/MRC_50_ values are in concordance with the numerical structural parameter counterparts in the Table 2. This also indicates that the individual structural parameters or their combinations are specific and the organism *H.e.* can be successful employed as a suitable test-system for further toxicity determinations.

## 5. Conclusions and Outlook

There is already wider recognition of the problem posed by the ever growing number of available chemicals with no tested toxicity in junction with the increased costs and limited time available for testing before entering mainstream production or they are dispersed into the environment. Therefore, the demand for developing *in silico* tools for providing the associated computed activities from benchmark measurements and individuated molecular fragment toxicities naturally appears; such studies should provide correlation paths regarding how the given toxicants may act on various cells or species. In moving towards such complex computational techniques for species and inter-species toxicity assignment the present work combines the Köln-*ESIP* and Timişoara-Spectral-SAR models in a unified computational activity-correlation framework.

The Köln model for estimation of a compound toxicity is based on the experimental measurement expressing the direct action of chemicals on the *H.e.* organism so that the structural influence parameters are reflected by the degree of metamorphosis itself. As such, the calculation of the structural parameters is absolute necessary for correctly evaluation and interpretation of the evolution of M(easured) and C(omputed) values.

The present work evidences relatively simple rules in respect to relationships with structure and reactivity: the efficacy of aliphatic alcohols increases with the number of the C atoms (a phenomenon characterized through the structural parameter logP) and diminishes with the appearance of new alcoholic groups (a phenomenon widely reflected through POL and E_tot_); the influence of the amino-group for aliphatic amines is comparatively predominant to the relative extent of the hydrocarbon chain [5]; the influence of the first methyl in the phenol case is negligible; the steric influence of isopropyl-radicals on the phenolic active centre for 2,6-derivative is stronger (by increased POL value) as in the case of the *t*-butyl substituent (while logP and POL values are diminished), etc.

In principle, the toxicity represents the synergetic effect of the three structural influences: hydrophobicity, electrostatic and steric molecular control on receptor binding. The efficiency difference through derivatives with closer parameters as those with 1,2-, 1,4- and 1,2,3-hydroxy groups can be interpreted through a particular electronic mechanism [6]; on the other hand the toxicity efficiency difference through 1,4-dihydroxybenzene (electronic) and 4-(3’,5’-dimethyl-3’-heptyl)phenol (steric) is well reflected through computed parameter’s numeric values.

Finally, the Timişoara Spectral-SAR analysis offers the correlation models and the end-points’ paths either for *H.e.* species as well for other four different organisms, with which the toxicity may be inter-changed by means of molecular structural mechanisms of action induced by certain common (or under testing) chemicals.

Besides the fact the Spectral-SAR algorithm was previously proven as being superior to the fashioned statistical approach in solving the paradoxical dichotomies various statistical indices produce when considered together [24], it advances a reliable method of identifying which structural molecular parameter is more influential across multiple possible paths of activation of a bio- or ecotoxicological response, thus furnishing a useful computational mechanistic molecular method in QSAR studies [29]. Then, when combined with *ESIP* algorithm a complex inter-molecular/inter-species toxicological transfer picture is provided.

However, the correlation maps depend on the algebraic or statistical way of modeling the action, *i.e.*, by assuming the chemical-biological interaction driven by the intensity norm (relating algebraic vectorial picture) or average (relating statistical dispersive picture) in ligand-receptor specific binding. At this point the algebraic vs. statistic issue remains open for further investigations by comparative single- and inter- species activities.

## Figures and Tables

**Table 1.** The vectorial (molecular) descriptors in a Spectral-SAR analysis represented as states, within the Hilbert *N*-dimensional space of investigated molecules.

***Activity***	***Structural predictor variables***					

|*Y_OBS_*_(_*_ERVED_*_)_〉	|*X*_0_〉	|*X*_1_〉	***…***	|*X_k_*〉	***…***	|*X_M_*〉
*y*_1-_*_OBS_*	1	*x*_11_	***…***	*x*_1_*_k_*	***…***	*x*_1_*_M_*
*y*_2-_*_OBS_*	1	*x*_21_	***…***	*x*_2_*_k_*	***…***	*x*_2_*_M_*
***…***	***…***	***…***	***…***	***…***	***…***	***…***
*y_N-OBS_*	1	*x_N_*_1_	***…***	*x_Nk_*	***…***	*x_NM_*

**Table 2.** The measured Mlog(1/MRC50) and ESIP-computed Clog(1/MRC50) toxicities for *Hydractinia echinata* and other organisms: for compounds nos. 2–7 from Ref. [5], for compounds nos. 13–21 from Ref. [6], new data for the rest; the Hansch molecular parameters as hydrophobicity (LogP), polarizability (POL) and the steric optimized total energy (E_tot_) were computed by HyperChem environment [26].

**No.**	**Compound**	***Species Toxicities* |Y>**										***Structural parameters***		

		***Hydractinia echinata***		***Tetrahymena pyriformis***		***Pimephales promelas***		***Vibrio fischeri***		***Daphnia magna***		|*X*_1_> **=Log P**	|*X*_2_> **=POL (A^3^)**	|*X*_3_>=E**tot (kcal/mol)**

		**Mlog**	**Clog**	**Mlog**	**Clog**	**Mlog**	**Clog**	**Mlog**	**Clog**	**Mlog**	**Clog**			

1	Water	−1.23	−0.91									−0.51	1.41	−8038.2
2	Methanol	−0.22	−0.41	0.33	0.15	0.04	0.24					−0.27	3.25	−11622.9
3	Ethanol	0.02	0.09	0.59	0.60	0.51	0.74	0.11	1.11	0.93	0.25	0.08	5.08	−15215.4
4	1-Butanol	0.99	1.08	1.48	1.50	1.63	1.73	1.34	2.18	1.57	1.68	0.94	8.75	−22402.8
5	1,2,3-Propanetriol	0.34	0.37									−1.08	8.19	−33600.
6	Triphenylmethanol	5.69	5.27									4.87	32.23	−68532.5
7	1,10-Diaminodecane	3.26	2.91									1.48	21.83	−46754.2
8	2-Benzylpyridine	3.75	3.46	3.41	4.85							3.53	21.22	−43675.3
9	4-Benzylpyridine	4.08	3.46	3.68	4.85							3.75	21.22	−43676.8
10	4-Phenylpyridin	4.13	3.46	3.66	3.46	3.98	3.81	4.91	4.84			3.35	19.38	−40083.1
11	4-Toluidine	2.85	2.02	2.98	2.81	3.43	3.26					1.73	13.62	−28300.3
12	1,2-Dichlorobenzene	3.04	3.45	4.00	3.66	4.19	4.17					3.08	14.29	−36217.2
13	Phenol(3,15/2,66/2,85)*	2.89*	2.87	2.79	2.58	3.41	3.21	3.42	3.68	3.32	3.32	1.76	11.07	−27003.1
14	2-Methylphenol(3,18/3,24)*	3.21*	2.82	2.72	2.58	3.77	3.21	3.75	3.68	3.64	3.32	2.23	12.91	−30596.6
15	2,4,6-Trimethyphenol(3,19/4,00)*	3.60*	3.82	3.42	3.48	4.02	4.21	4.08	4.75	4.49	4.75	3.16	16.58	−37783.7
16	1,2-Dihydroxibenzene	5.11	5.11	3.75	3.47	4.08	4.08	3.54	3.54	4.68	4.24	1.48	11.71	−34396.4
17	2-Methoxyphenol(2,89/2,77)*	2.83*	3.28	2.49	2.54				3.29			1.51	13.54	−37974.4
18	1,4-Dihydroxybenzene(6,14/6,06)*	6.10*	6.10	3.47	3.59	6.40	6.40	6.42	6.42			1.48	11.71	−34395.8
19	t-Butylhydroquinone(5,05/5,00)*	5.30*	7.60		4.94		7.78		8.03			3.11	19.05	−48758.1
20	1,2,3-Trihydroxibenzene	5.15	5.15	3.85	3.65							1.19	12.35	−41789.9
21	4(3',5'-dimethyl--3'-heptyl) phenol	7.65	6.81									4.87	25.75	−55742.
22	4-Chlorophenol	3.25	3.04	3.55	3.56	4.18	3.66	4.19	3.88	4.13	3.95	2.28	13	−35307.6
23	2,6-Diisopropylphenol	3.73	5.31		4.82		5.21		6.36		6.90	4.15	22.08	−48554.7
24	2-Aminophenol	3.15	3.04	3.94	2.93							0.98	12.42	−32098.6
25	2,4,6-Trinitrophenol	2.92	2.99	2.84				2.63	3.77			−4.17	16.59	−84472.1
26	Chloranil	5.15	−									1.12	18.51	−66928.2
27	Chloranilic acid	3.40	2.99									−0.48	15.93	−65113.6
28	4-Methoxyazobenzene	5.20	3.70									4.10	24.63	−59069.5

* The M* values represents the experimental results accomplished by different time interval with different generations of *H.e*. These results clearly prove the reproducibility of the test-system.

**Table 3.** Mlog-Spectral Spectral-SAR results employing the molecular parameters and the *Hydractinia echinata* (*H.e.*), *Tetrahymena pyriformis* (*T.p.*), *Pimephales promelas* (*P.p.*), *Vibrio fisheri* (*V.f.*), and *Daphnia magna* (*D.m.*) toxicities of Table 2; the models are characterized either by algebraic norms and correlation factors (*RA*), computed upon the Equations (6) and (8), and by Pearson statistical correlation (*R*) of Equation (7), for all possible mono-, bi-, and all- end-points, respectively. The referential algebraic norms of the considered species were estimated with the aid of Equation (6) from the Mlog input toxicity data of Table 2 as: ║|Y_H.e._>║ = 20.8547, ║|Y_T.p._>║ = 13.2774, ║|Y_P.p._>║ = 12.8515, ║|Y_V.f._>║ = 12.1055, ║|Y_D.m._>║ = 9.31242.

***Mlog Model***	***Species***	***Spectral-SAR Activity Equation***	***Spectral-SAR Norm***	***RA (Algebraic)***	***R (Statistic)***
|1>	*H.e.*	|*Y_H.e_*^|1>^〉 = 2.348 + 0.595 |LogP>	19.0572	0.9138	0.5912
	*T.p.*	|*Y_T.p_*^|1>^〉 = 2.526+0.267 |LogP>	12.642	0.9521	0.4446
	*P.p.*	|*Y_P.p_*^|1>^〉 = 1.402 +1.071 |LogP>	12.1481	0.9453	0.6972
	*V.f.*	|*Y_V.f_*^|1>^〉 = 2.981 + 0.364 |LogP>	11.1235	0.9189	0.4396
	*D.m.*	|*Y_D.m_*^|1>^〉 = 1.192 + 1.208 |LogP>	9.09749	0.9769	0.8300

|2>	*H.e.*	|*Y_H.e_*^|2>^〉 = 0.022 + 0.221 |POL>	19.7048	0.9449	0.7597
	*T.p.*	|*Y_T.p_*^|2>^〉 = 0.72 + 0.168 |POL>	12.9074	0.9721	0.7267
	*P.p.*	|*Y_P.p_*^|2>^〉 = −0.109 + 0.29 |POL>	12.2254	0.9513	0.7357
	*V.f.*	|*Y_V.f_*^|2>^〉 = 0.121 + 0.262 |POL>	11.3472	0.9374	0.6092
	*D.m.*	|*Y_D.m_*^|2>^〉 = −0.759 + 0.355 |POL>	9.16099	0.9837	0.8832

|3>	*H.e.*	|*Y_H.e._*^|3>^〉 = 0.433 –0.00007 |E_tot_ >	19.2139	0.9213	0.6355
	*T.p.*	|*Y_T.p_*^|3>^〉 = 1.669 –3.6·10^−5^ |E_tot_ >	12.6819	0.9551	0.4969
	*P.p.*	|*Y_P.p._*^|3>^〉 = –1.767 – 1.7·10^−4^ |E_tot_ >	12.5439	0.9761	0.8785
	*V.f.*	|*Y_V.f._*^|3>^〉 = 2.755 – 1.89·10^−5^|E_tot_ >	10.926	0.9026	0.1982
	*D.m.*	|*Y_D.m_*^|3>^〉 = −1.826 –1.75·10^−4^ |E_tot_ >	9.26686	0.9951	0.9662

|1,2>	*H.e.*	|*Y_H.e_*^|1,2>^〉 = 0.206 + 0.163|LogP> +0.19 |POL>	19.7462	0.9468	0.7694
	*T.p.*	|*Y_T.p_*^|1,2>^〉 = 0.784 +0.093|LogP> +0.152 |POL>	12.9228	0.9733	0.7398
	*P.p.*	|*Y_P.p_*^|1,2>^〉 = −0.324 –0.191|LogP> + 0.337 |POL>	12.2271	0.9514	0.7365
	*V.f.*	|*Y_V.f._*^|1,2>^〉 = −0.018 + 0.307|LogP> + 0.242 |POL>	11.5146	0.9512	0.7116

|1,3>	*H.e.*	|*Y_H.e_*^|1,3>^〉 = −0.296 + 0.541|LogP> – 0.00007 |E_tot_ >	20.0182	0.9599	0.8307
	*T.p.*	|*Y_T.p._*^|1,3>^〉 = 0.433 + 0.413| LogP> – 5.27·10^−5^ |E_tot_ >	13.018	0.9805	0.8171
	*P.p.*	|*Y_P.p_*^|1,3>^〉 = −3.541 −1.061|LogP> −2.96·10^−4^ |E_tot_ >	12.646	0.9840	0.9203
	*V.f.*	|*Y_V.f._*^|1,3>^〉 = −0.512 +0.82 |LogP> −8.1·10^−5^ |E_tot_ >	11.6329	0.9610	0.7767

|2,3>	*H.e.*	|*Y_H.e._*^|2,3>^〉 = −0.134 + 0.193|POL> – 0.00001 |E_tot_ >	19.7224	0.9457	0.7638
	*T.p.*	|*Y_T.p._*^|2,3>^〉 = 0.704 +0.163 |POL> – 2.18·10^−6^ |E_tot_ >	12.9078	0.9722	0.7270
	*P.p.*	|*Y_P.p._*^|2,3>^〉 = −2.269 −0.262|POL> −2.94·10^−4^ |E_tot_ >	12.6208	0.9820	0.9101
	*V.f.*	|*Y_V.f._*^|2,3>^〉 = 0.082 + 0.36 |POL> +3.35〉10^−5^ |E_tot_ >	11.4347	0.9446	0.6645

{|1,2,3>}	*H.e.*	|*Y_H.e_*^{|1,2,3>}^〉 = −0.259 + 0.979|LogP> −0.214|POL> −0.00013|E_tot_ >	20.1085	0.9642	0.8502
	*T.p.*	|*Y_T.p_*^{|1,2,3>}^〉 = 0.456 + 0.773 |LogP> −0.185|POL> −0.00011|E_tot_ >	13.0541	0.9832	0.8447

**Table 4.** The same type of Spectral-SAR models as those of Table 3, here for Clog data of Table 2. The referential algebraic norms of the considered species were estimated with the Equation (6) from the Clog input toxicity data of Table 2 as: ║|Y_H.e._>║ = 20.1051, ║|Y_T.p._>║ = 14.8984, ║|Y_P.p._>║ = 15.5929, ║|Y_V.f._>║ = 16.6682, ║|Y_D.m._>║ = 11.3438.

***Clog Model***	***Species***	***Spectral-SAR Activity Equation***	***Spectral-SAR Norm***	***RA (Algebraic)***	***R (Statistic)***
|1>	*H.e.*	|*Y_H.e_*^|1>^〉 = 2.242 + 0.583 |LogP>	18.1498	0.9027	0.5744
	*T.p.*	|*Y_T.p_*^|1>^〉 = 1.248+ 0.919 |LogP>	14.6075	0.9805	0.8572
	*P.p.*	|*Y_P.p_*^|1>^〉 = 1.436 + 1.107 |LogP>	14.7182	0.9439	0.7011
	*V.f.*	|*Y_V.f_*^|1>^〉 = 3.598 + 0.41 |LogP>	15.6785	0.9406	0.4605
	*D.m.*	|*Y_D.m_*^|1>^〉 = 0.57 + 1.483 |LogP>	11.2079	0.9880	0.9432

|2>	*H.e.*	|*Y_H.e_*^|2>^〉 = 0.242 + 0.201 |POL>	18.5655	0.9234	0.6831
	*T.p.*	|*Y_T.p_*^|2>^〉 = –0.118 + 0.237 |POL>	14.7122	0.9875	0.9108
	*P.p.*	|*Y_P.p_*^|2>^〉 = –0.092 + 0.29 |POL>	14.871	0.9537	0.7604
	*V.f.*	|*Y_V.f_*^|2>^〉 = 0.111 + 0.298 |POL>	16.1385	0.9682	0.7565
	*D.m.*	|*Y_D.m_*^|2>^〉 = –1.241 + 0.379 |POL>	11.2605	0.9927	0.9655

|3>	*H.e.*	|*Y_H.e_*^|3>^〉 = 0.518 – 0.00007 |E_tot_ >	18.16	0.9033	0.5773
	*T.p.*	|*Y_T.p_*^|3>^〉 = –1.176 –1.27·10^−4^ |E_tot_ >	14.8013	0.9935	0.9544
	*P.p.*	|*Y_P.p_*^|3>^〉 = –1.597 – 1.64·10^−4^ |E_tot_ >	15.2359	0.9771	0.8882
	*V.f.*	|*Y_V.f._*^|3>^〉 = 2.546 – 4.51·10^−5^ |E_tot_ >	15.6221	0.9372	0.4106
	*D.m.*	|*Y_D.m_*^|3>^〉 = –2.546 –1.94·10^−4^ |E_tot_ >	11.3184	0.9978	0.9896

|1,2>	*H.e.*	|*Y_H.e_*^|1,2>^〉 = 0.488 + 0.217 |LogP> + 0.16 |POL>	18.6415	0.9272	0.7014
	*T.p.*	|*Y_T.p_*^|1,2>^〉 = –0.208 – 0.081|LogP> + 0.255 |POL>	14.7128	0.9875	0.9112
	*P.p.*	|*Y_P.p._*^|1,2>^〉 = –1.038 – 0.931|LogP> + 0.509 |POL>	14.908	0.9561	0.7742
	*V.f.*	|*Y_V.f._*^|1,2>^〉 = 0.228 + 0.188 |LogP> + 0.268 |POL>	16.187	0.9711	0.7816

|1,3>	*H.e.*	|*Y_H.e_*^|1,3>^〉 = –0.134 + 0.522 |LogP> – 0.00006 |E_tot_ >	18.9449	0.9423	0.7708
	*T.p.*	|*Y_T.p._*^|1,3>^〉 = –0.859 + 0.219 |LogP> – 1.04·10^−4^ |E_tot_ >	14.8152	0.9944	0.9611
	*P.p.*	|*Y_P.p_*^|1,3>^〉 = –3.524 – 1.327|LogP>– 3.1·10^−4^ |E_tot_ >	15.4088	0.9882	0.9437
	*V.f.*	|*Y_V.f._*^|1,3>^〉 = –0.12 + 0.713 |LogP> – 8.42·10^−5^ |E_tot_ >	16.2777	0.9766	0.8267

|2,3>	*H.e.*	|*Y_H.e_*^|2,3>^〉 = 0.093 + 0.175 |POL> – 0.00001 |E_tot_ >	18.5804	0.9242	0.6868
	*T.p.*	|*Y_T.p._*^|2,3>^〉 = –1.045 + 0.062 |POL> – 9.77·10^−5^|E_tot_ >	14.8118	0.9942	0.9594
	*P.p.*	|*Y_P.p_*^|2,3>^〉 = –2.243 –0.355 |POL> –3.28·10^−4^ |E_tot_ >	15.3717	0.9858	0.9320
	*V.f.*	|*Y_V.f_*^|2,3>^〉 = 0.2 + 0.337 |POL> + 1.65·10^−5^ |E_tot_ >	16.1548	0.9692	0.7650

{|1,2,3>}	*H.e.*	|*Y_H.e_*^{|1,2,3>}^〉 = –0.166 + 1.229|LogP> – 0.351|POL> –0.00017|E_tot_ >	19.1684	0.9534	0.8188
	*T.p.*	|*Y_T.p_*^{|1,2,3>}^〉 = –0.871 + 0.199|LogP> + 0.008|POL> –0.0001|E_tot_ >	14.8153	0.9944	0.9611

**Table 5.** Synopsis of the statistic and algebraic values of paths connecting the Spectral-SAR models for *Hydractinia echinata* (*H.e.*) and *Tetrahymena pyriformis* (*T.p.*) in the Mlog/Clog and algebraic/statistical computational frames of Tables 3 and 4. The primary, secondary and tertiary - the so called *alpha* (α), *beta* (β) and *gamma* (γ) - paths are indicated according to the least path principle in spectral norm-correlation space, respectively.

Species Method Paths	*H.e.*				*T.p.*			

	**Mlog**		**CLog**		**Mlog**		**Clog**	

	*Algebraic*	*Statistic*	*Algebraic*	*Statistic*	*Algebraic*	*Statistic*	*Algebraic*	*Statistic*
|1>→|1,2>→|1,2,3>	1.05246	***1.08283^γ^***	1.01981	***1.0476^γ^***	0.41325	***0.575439^γ^***	0.208278	0.232353
|1>→|1,3>→|1,3,2>	**1.05246^γ^**	1.08273	**1.01981^γ^**	1.04754	**0.41325^γ^**	0.574673	**0.208278^γ^**	***0.232342^γ^***
|1>→|2,3>→|1,2,3>	1.05246	1.08284	1.01981	1.0476	0.41325	0.575534	0.208278	0.232342

|2>→|1,2>→|2,1,3>	0.404191	0.413755	0.603637	0.61798	0.147067	0.188257	**0.103313^β^**	***0.114674^β^***
|2>→|1,3>→|2,1,3>	0.404191	0.413756	0.603637	0.617987	0.147067	0.188265	0.103313	0.114674
|2>→|2,3>→|2,3,1>	**0.404191^α^**	***0.413754*^*α*^**	**0.603637^α^**	***0.617972*^*α*^**	**0.147067^α^**	***0.188246*^*α*^**	0.103313	0.114674

|3>→|1,2>→|3,1,2>	**0.89559^β^**	0.920041	**1.00961^β^**	1.03703	**0.373261^β^**	0.510175	0.191347	0.212443
|3>→|1,3>→|3,1,2>	0.89559	***0.919987*^*β*^**	1.00961	***1.03697*^*β*^**	0.373261	***0.509664*^*β*^**	0.0140336	0.0155182
|3>→|2,3>→|3,2,1>	0.89559	0.920044	1.00961	1.03703	0.373261	0.510232	**0.0140336^α^**	***0.0155182^α^***

**Table 6.** The same type of information and analysis as in Table 5, here for *Pimephales promelas* (*P.p.*) and *Vibrio fisheri* (*V.f.*) species.

Species Method Paths	*P.p.*				*V.f.*			

	**Mlog**		**CLog**		**Mlog**		**Clog**	

	*Algebraic*	*Statistic*	*Algebraic*	*Statistic*	*Algebraic*	*Statistic*	*Algebraic*	*Statistic*
|1>→|1,2>	0.0792073	0.0881801	0.190201	0.2034	**0.392451^β^**	***0.476398^β^***	**0.509418^β^**	***0.601392^β^***
|1>→|1,3>	**0.499343^γ^**	***0.545515^γ^***	**0.692013^γ^**	***0.731962^γ^***	0.511148	0.61083	0.600329	0.702271
|1>→|2,3>	0.474093	0.518389	0.654817	0.69307	0.312213	0.383883	0.477152	0.565317

|2>→|1,2>	**0.00166893^α^**	***0.0018496^α^***	**0.0371086^α^**	***0.0395137^α^***	0.167993	0.196303	0.0485269	0.0545366
|2>→|1,3>	0.421805	0.459267	0.538921	0.568184	0.28669	0.331225	0.139438	0.155864
|2>→|2,3>	0.396554	0.432134	0.501725	0.529282	**0.0877552^α^**	***0.103489^α^***	**0.0162612^α^**	***0.0183134^α^***

|3>→|1,2>	0.3177	0.347114	0.328541	0.347126	0.590616	0.781086	0.565916	0.675813
|3>→|1,3>	0.102435	0.11035	0.173271	0.181596	**0.709313^γ^**	***0.913454^γ^***	**0.656827^γ^**	***0.776511^γ^***
|3>→|2,3>	**0.0771849^β^**	***0.0832042^β^***	**0.136075^β^**	***0.142683^β^***	0.510379	0.690032	0.53365	0.639803

**Table 7.** Synopsis of the interspecies minimum paths and the associated ordered endpoints for each of the Mlog/Clog-algebraic/statistic modes of computations abstracted from the Tables 5 and 6.

**Computational Modes**		**Minimum Interspecies Paths**			**Ordered Endpoints**
		***alpha***	***beta***	***gamma***	
***Algebraic***	***Mlog***	*α_P.p._*	*β_P.p._*	*γ_T.p._*	
		|2>→|1,2>	|3>→|2,3>	|1>→|1,3>	|2>→|3>→|1>→|1,2>→|2,3>→|1,3>→{|1,2,3>}

	***Clog***	*α_T.p._*	*β_T.p._*	*γ_T.p._*	
		|3>→|2,3>	|2>→|1,2>	|1>→|1,3>	|3>→|2>→|1>→|2,3>→|1,2>→|1,3>→{|1,2,3>}

***Statistic***	***Mlog***	*α_P.p._*	*β_P.p._*	*γ_P.p._*	
		|2>→|1,2>	|3>→|2,3>	|1>→|1,3>	|2>→|3>→|1>→|1,2>→|2,3>→|1,3>→{|1,2,3>}

	***Clog***	*α_T.p._*	*β_T.p._*	*γ_T.p._*	
		|3>→|2,3>	|2>→|1,2>	|1>→|1,3>	|3>→|2>→|1>→|2,3>→|1,2>→|1,3>→{|1,2,3>}

## Appendix

### A1. Vectorial Scalar Product, Norms, and Cauchy-Schwarz Inequality

Given two vectors:

(A1) $$|u\rangle =|u_{1},\;u_{2},\ldots ,u_{n}\rangle ,\;|v\rangle =|v_{1},v_{2},\ldots ,v_{n}\rangle$$

their scalar (or dot) product writes as:

(A2) 〈u|v〉=def∑i=1nuivi=u1v1+u2v2+........+unvn

Since the *self* scalar product looks like:

(A3) $$\langle u|u\rangle =\sum_{i=1}^{n}u_{i}^{2}$$

one may introduce the so called norm (or length) of the vector by definition:

(A4) $$\left|\left||u\rangle \right|\right|=\sqrt{\langle u|u\rangle }=\sqrt{\sum_{i=1}^{n}u_{i}^{2}}$$

The length property of the vectorial norm may be easily visualized through computing the modulus of an arbitrary 3D vector, say |*r*〉 = |*u*_1_,*u*_2_,*u*_3_〉:

(A5) $$\left|\vec{r}\right|=\sqrt{u_{1}^{2}+u_{2}^{2}+u_{3}^{2}}=\sqrt{\langle u_{1},u_{2},u_{3}|u_{1},u_{2},u_{3}\rangle }=\sqrt{\langle r|r\rangle }=\left|\left||r\rangle \right|\right|$$

Consequently, the distance between two vectors may be written in terms of their difference norm as:

(A6) $$d(|u\rangle ,|v\rangle )=\left|\left||u\rangle -|v\rangle \right|\right|=\left|\left||u-v\rangle \right|\right|=\sqrt{\langle u-v|u-v\rangle }=\sqrt{\sum_{i=1}^{n}{\left(u_{i}-v_{i}\right)}^{2}}$$

From Equation (A6), but also from the fact that the self-scalar product is positively defined, see Equation (A4), the distributivity and commutativity properties of scalar product may be employed for any real parameter, *t*∈ ℜ, towards equivalent expressions:

(A7) $$\begin{array}{c} \langle u-tv|u-tv\rangle \geq 0,\;t\in ℜ\\ \Leftrightarrow (\langle u|-\langle v|t)(|u\rangle -t|v\rangle )\geq 0\\ \Leftrightarrow \langle v|v\rangle t^{2}-2\langle u|v\rangle t+\langle u|u\rangle \geq 0 \end{array}$$

The last inequality says that the left sided second order equation has no solution or has two equal solutions; such condition is fulfilled when its discriminator is less or equal with zero, respectively, leading with the famous *Cauchy-Schwartz inequality*:

(A8) $${\langle u|v\rangle }^{2}\leq \langle u|u\rangle \langle v|v\rangle$$

which may be rewritten as:

(A9) $$\sum_{i=1}^{n}u_{i}v_{i}\leq \sqrt{\sum_{i=1}^{n}u_{i}^{2}}\sqrt{\sum_{i=1}^{n}v_{i}^{2}}$$

or in more formal way as:

(A10) |〈u|v〉|≤|||u〉||⋅|||v〉||

The Cauchy-Schwartz inequality is successfully used in probability theory, variance theory and correlation factors. An actual application is in following given as well.

### A2. Algebraic Correlation Factor

One starts with the simple connection between the observed, predicted and error vectors of Equation (1a), however specialized on their individual elements:

(A11) $$Y_{i-\text{OBS}}=Y_{i-\text{PRED}}+pe_{i}$$

where “*pe*” here stays as the abbreviation for “prediction error”.

Then, while squaring relation (A11):

(A12) $$Y_{i-\text{OBS}}^{2}=Y_{i-\text{PRED}}^{2}+pe_{i}^{2}+2Y_{i-\text{PRED}}\cdot pe_{i}$$

and summing for all working *N*-molecules (of Table 1):

(A13) $$\sum_{i=1}^{N}Y_{i-\text{OBS}}^{2}=\sum_{i=1}^{N}Y_{i-\text{PRED}}^{2}+\sum_{i=1}^{N}pe_{i}^{2}+2\sum_{i=1}^{N}Y_{i-\text{PRED}}\cdot pe_{i}$$

the last relation simplifies to:

(A14) $$\sum_{i=1}^{N}Y_{i-\text{OBS}}^{2}=\sum_{i=1}^{N}Y_{i-\text{PRED}}^{2}+\sum_{i=1}^{N}pe_{i}^{2}$$

based on applying of scalar product definition (A2) and of prediction error orthogonalization condition (1c) for the last term of (A13):

(A15) $$\sum_{i=1}^{N}Y_{i-\text{PRED}}\cdot pe_{i}=\langle Y_{\text{PRED}}|pe\rangle =0$$

Now, substituting the prediction error values of (A11) in (A14) one equivalently yields:

(A16) $$\begin{array}{l} \sum_{i=1}^{N}Y_{i-\text{OBS}}^{2}=\sum_{i=1}^{N}Y_{i-\text{PRED}}^{2}+\sum_{i=1}^{N}(Y_{i-\text{OBS}}-Y_{i-\text{PRED}}{)}^{2}\\ \;\;\;\;\;\;\;\;\;\;\;\;\;\Leftrightarrow \sum_{i=1}^{N}Y_{i-\text{PRED}}^{2}=\sum_{i=1}^{N}Y_{i-\text{OBS}}\cdot Y_{i-\text{PRED}} \end{array}$$

which further rewrites, recalling the norm and scalar product definitions of Equations (A4) and (A2), respectively, as:

(A17) $${\left|\left||Y_{\text{PRED}}\rangle \right|\right|}^{2}=\langle Y_{\text{OBS}}|Y_{\text{PRED}}\rangle$$

Finally, the Cauchy-Schwarz form (A10) is employed on the right side term of (A17), noting that the observed and predicted activities are of the same nature for a given molecule – *i.e.*, either both positive or both negative – thus providing their scalar product as positively defined; yet this is certified also by the relation (A17) viewed as a result *per se*; nevertheless, through considering Cauchy-Schwarz prescription, the relation (A17) immediately transforms into inequality:

(A18) $${\left|\left||Y_{\text{PRED}}\rangle \right|\right|}^{2}\leq \left|\left||Y_{\text{OBS}}\rangle \right|\right|\cdot \left|\left||Y_{\text{PRED}}\rangle \right|\right|$$

leaving with the predicted-observed norms’ hierarchy:

(A19) $$\left|\left||Y_{\text{PRED}}\rangle \right|\right|\leq \left|\left||Y_{\text{OBS}}\rangle \right|\right|$$

Relation (A19) guarantees the *consistent probability definition* for the introduced *algebraic correlation factor* of Equation (8):

(A20) $$r_{\text{ALGEBRAIC}}=\frac{\left|\left||Y_{\text{PRED}}\right|\right|}{\left|\left||Y_{\text{OBS}}\right|\right|}\equiv RA\leq 1$$
